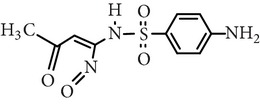# Degradation Effect of Sulfa Antibiotics by Potassium Ferrate Combined with Ultrasound (Fe(VI)-US)

**DOI:** 10.1155/2015/169215

**Published:** 2015-08-11

**Authors:** Kejia Zhang, Zhang Luo, Tuqiao Zhang, Naiyun Gao, Yan Ma

**Affiliations:** ^1^College of Civil Engineering and Architecture, Zhejiang University, Hangzhou 310058, China; ^2^Key Laboratory of Drinking Water Safety and Distribution Technology of Zhejiang Province, Zhejiang University, Hangzhou 310058, China; ^3^State Key Laboratory of Pollution Control and Resource Reuse, Tongji University, Shanghai 200092, China; ^4^Shanghai Urban Water Resources Development and Utilization National Engineering Center Co. Ltd., Shanghai 200082, China

## Abstract

Sulfa antibiotics are a family of typical broad-spectrum antibiotics, which have become one of the most frequently detected antibiotics in water, posing a great threat to human health and ecosystem. Potassium ferrate is a new type of high-efficiency multifunctional water treatment agent, collecting the effects of oxidation, adsorption, flocculation, coagulation, sterilization, and deodorization. Performance and mechanism of degradation of typical broad-spectrum antibiotics by Fe(VI)-US were further studied, investigating the degradation effect of sulfa antibiotics by single ultrasound, single potassium ferrate, and potassium ferrate-ultrasound (Fe(VI)-US). It was found that Fe(VI)-US technology had a significant role in promoting the degradation of sulfa antibiotics via orthogonal experiments. Factors evaluated included sulfa antibiotics type, pH value, potassium ferrate dosage, ultrasonic frequency, and ultrasonic power, with the pH value and potassium ferrate dosage being affected most significantly. One reason for synergy facilitating the degradation is the common oxidation of potassium ferrate and ultrasound, and the other is that Fe(III) produced promotes the degradation rate. According to the product analysis and degradation pathways of three sulfa antibiotics, ferrate-sonication sulfa antibiotics are removed by hydroxyl radical oxidation.

## 1. Introduction

Due to the toxicity of antibiotics and their wide usage and even abuse in the treatment of human and poultry, beasts of infectious diseases, antibiotics, have become a hot issue in water treatment nowadays. Recently, a certain concentration of antibiotic residues has been detected in surface water, groundwater, drinking water, sludge and soil, and some other environmental media [1, 2]. Ninety kinds of organic pollutants were detected in 139 rivers from more than 30 states of USA, including pesticides, pharmaceuticals, veterinary drugs, and hormones [3]. Samples were taken from the 7 sites and 15 substations of agricultural watershed of the USA, and the existence of antibiotics in water samples was measured [4]. A certain concentration of antibiotics was detected in municipal wastewater, farmland soil, surface water, and even drinking water in Germany [5]. It was found that they contained high concentration of antibiotics in the sediments of fish farming area of Wujiangdu reservoir (China), with chloramphenicol (CAP) 5–37 *μ*g·kg^−1^, oxytetracycline (OTC) 21–156 *μ*g·kg^−1^, tetracycline (TC) 84–248 *μ*g·kg^−1^, and chlortetracycline (CTC) 42–90 *μ*g·kg^−1^ [6]. In Guiyang (China), antibiotics were also found in city life sewage, with CAP 1.8–4.3 *μ*g·L^−1^, OTC 5–8 *μ*g·L^−1^, TC 4.4–10 *μ*g·L^−1^, and CTC 0–2.2 *μ*g·L^−1^.

As typical broad-spectrum antibiotics, sulfa antibiotics are commonly used in clinical treatment, animal husbandry, and aquaculture. With the rapid development of Chinese animal husbandry, the usage of sulfa antibiotics as veterinary drugs and fishery drugs increased. Some studies analyzed the content and distribution of four kinds of sulfa antibiotics in the dung of 20 scale farms in Guangdong province (China); the results showed that sulfa compounds in dung was 1925.9–13399.5 *μ*g·kg^−1^, mainly sulfamerazine and sulfamethoxazole. Sulfa antibiotics detection rate in cow dung was more than 90%, the content of which was 1039.4–15930.3 *μ*g·kg^−1^, mainly sulfamethoxazole and sulfamerazine [7]. Sulfa antibiotics in farm manure discharged into waters with rainwater runoff and farm wastewater in abundance, which have become one of the most frequently detected antibiotics in water, and their distributions in the environment were not optimistic [3]. From 2006 to 2007, 19 water companies in USA detected raw water, treated water, and pipeline water, the most commonly detected compounds reached up to 11 kinds, and sulfa antibiotics (sulfamethoxazole) was one of them [8]. Twenty-two kinds of antibiotics were detected in the lake of Baiyangdian (China), among which sulfa antibiotics were the most widely distributed and were of the highest levels; the concentration reached 0.86–1563 ng·L^−1^ [9]. China's Pearl River Basin was studied by Xu [10], which included the residue of antibiotics both in Guangzhou and Shenzhen rivers, the content of sulfamethoxazole in Shenzhen river reaching 880 ng·L^−1^. Typical broad-spectrum sulfa antibiotics can be detected both in surface water and drinking water all over the world, which not only explains the serious material pollution now but also declares the fact that the conventional treatment process cannot remove sulfa antibiotics effectively. Fortunately, more and more attention has been given to the research of degradation of antibiotics in water environment. Generally speaking, there are physical, chemical, and biological treatment methods for the removal of antibiotics in wastewaters; all these methods can remove antibiotics effectively with high concentration of antibiotics. However, these methods cannot work well for drinking water sources with low levels of antibiotics. Therefore, much more energy should be devoted to study of the degradation of sulfa antibiotics in order to find out effective control methods, improving water quantity and ensuring water security.

The German chemical and physicist Georg Stahl first discovered and reported the ferrate in 1702; since then, the international research of ferrate has never stopped. In recent years, potassium ferrate has gained widespread attention in the field of water treatment for its strong oxidation. It is hexavalent (Fe(VI)) for iron element of potassium ferrate; potassium ferrate exists in the form of FeO_4_^2−^ in aqueous solution, having extremely strong oxidizing with redox potential +2.20 V and +0.72 V, respectively, in acidic and alkaline conditions [11]. Potassium ferrate have strong oxidizing under acid condition, the oxidation of which is much stronger than the common water treatment disinfectants such as chlorine, ozone, hydrogen peroxide, and chlorine dioxide [12].

Potassium ferrate oxidation has a strong selectivity for pollutants, and the oxidation rate and efficiency in different compounds are different. The removal rate of some refractory organics is not high when potassium ferrate is used alone, and potassium ferrate itself is easily decomposed at low pH value, affecting its oxidation efficiency. Therefore, there is a need to research potassium ferrate coupling technique for achieving better treatment effect. Potassium ferrate coupling technique is a hot topic and application direction in the study of potassium ferrate; the existing techniques mainly include potassium ferrate-aluminium, potassium ferrate-ozone, and potassium ferrate-photocatalysis.

This study took the typical broad-spectrum sulfa antibiotics as target pollutants, removed by Fe(VI)-US oxidation, studying different impacts of degradation effect of sulfadiazine, establishing the reaction kinetics model, investigating the degradation mechanism of sulfa antibiotics, and providing certain theoretical basis and technical support for drugs polluted water treatment.

## 2. Materials and Methods

### 2.1. Chemicals

Sulfadiazine (purity > 99%), sulfamerazine (purity > 99%), and sulfamethoxazole (purity > 99%) were all purchased from Sigma-Aldrich (USA); their physical and chemical properties (molecular formula, structural formula, molecular weight, ionization equilibrium constant pKa, etc.) were shown in Table 1. Oxidant potassium ferrate (purity > 90%) was also purchased from Sigma-Aldrich (USA). Buffer solution used in the experiment was made up of analytical grade of potassium hydroxide, dipotassium hydrogen phosphate, potassium dihydrogen phosphate, and potassium borate, with sodium thiosulfate being reaction terminators. Mobile phases of formic acid purchased from Fluka (Switzerland) and acetonitrile purchased from Sigma-Aldrich (USA) are chromatographic grade; formic acid solution was prepared from Milli-Q ultrapure water (18.2 Ω). Other chemicals were of analytical grade and above, and all were purchased from Sinopharm Chemical Reagent Co., Ltd. Water used in experiment was deionized water.

### 2.2. Experimental Apparatus and Methods

#### 2.2.1. Apparatus and Methods of Ferrate Oxidation

Degradation of sulfa antibiotics by Fe(VI) took place in 150 mL conical flask. The pH value of reaction solution was adjusted by buffer solution; add a certain amount of Fe(VI) into the reaction solution under the condition of magnetic stirring, take samples at scheduled time, and then add a small amount of 0.1 mol·L^−1^ sodium thiosulfate to terminate the reaction. Samples taken were analyzed after centrifugation at 6000 r/min for 10 min; all experiments were performed at room temperature (25 ± 2°C).

#### 2.2.2. Apparatus and Methods of Ultrasonic Reaction

**Figure 1:**
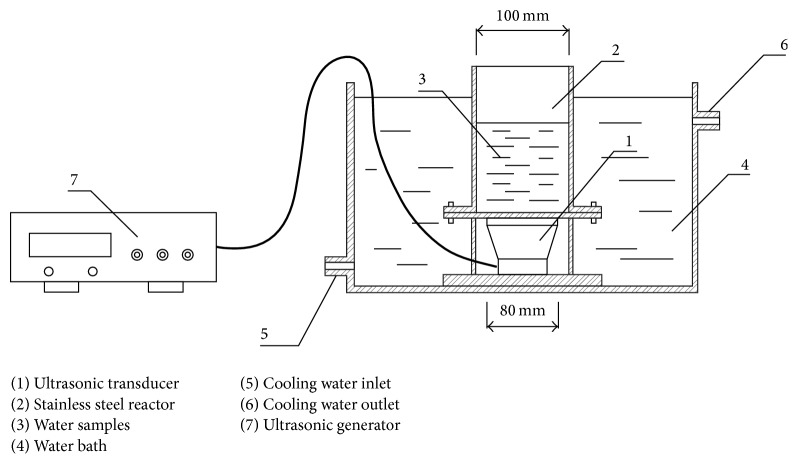
Schematic diagram of ultrasonic apparatus.

Ultrasonic reactor used was purchased from Shanghai Poly Fiber Ultrasound Equipment Co., Ltd., consisting of ultrasonic generator, ultrasonic transducer, and reaction vessel. There are four ultrasonic generators; the models are, respectively, HF-200 (ultrasonic frequency *f* = 223 kHz), HF-400 (*f* = 400 kHz), HF-600 (*f* = 600 kHz), and HF-800 (*f* = 800 kHz); ultrasonic power can be adjusted ranging from 30 to 100 W, with maximum power density 3 W·cm^−2^. Ultrasonic reaction was conducted in an open stainless steel cylinder (inner diameter Φ = 10.0 cm, *H* = 10.0 cm), the bottom of the cylinder was directly connected with the ultrasound transducer, and the joints were sealed with PTFE O-ring. Stainless steel cylinder was placed in a thermostatic water bath system (Figure 1), with the reaction temperature controlled at 25 ± 2°C.

The volume of reaction solution was 100 mL in all experiments, and the pH value was adjusted by buffer solution. Reaction was conducted in the stainless steel reactor, and the samples were collected at predetermined time intervals for the analysis of sulfa antibiotics. When performing Fe(VI)-US samples taken should be added to a small amount of 0.1 mol·L^−1^ sodium thiosulfate to terminate reaction, with the samples being analyzed after centrifugation.

### 2.3. Analytical Methods and Detecting Instrument

#### 2.3.1. Detection of Fe(VI) Concentration

Fe(VI) exists in the form of FeO_4_^2−^ when dissolved in water; the solution is purple, with a clear UV-visible spectrum; the absorption curve has been shown in appropriate concentration range (1.41 × 10^−5^–5.05 × 10^−4 ^mol·L^−1^) [13]; there is a linear relationship between concentration and absorbance of Fe(VI) solution; thus, the corresponding concentration can be obtained by measuring the absorbance of Fe(VI) solution. In experiment, absorbance of Fe(VI) solution was measured by UV/VIS spectrophotometer instrument; the concentration was calculated according to Bill-Lambert's law as follows:

(1) $$\begin{array}{c} A=\varepsilon bc, \end{array}$$

where *ε* is molar absorption coefficient, reflecting the solution absorption capacity of a wavelength of light, L·mol^−1^·cm^−1^; *b* is cuvette width, cm; *c* is analyte concentration, mol·L^−1^.

When cuvette width *b* is 1 cm, Bill Lambert's law is expressed in

(2) $$\begin{array}{c} A=\varepsilon c. \end{array}$$

Absorbance of 0.25 mmol·L^−1^ and 0.51 mmol·L^−1^ of potassium ferrate solution reached maximum at 510 nm wavelength. At the same time, it was documented that reaction products Fe(II) and Fe(III) phosphate would not interfere with the detection at 510 nm wavelength; thus, we can accurately track the Fe(VI) concentration change and adopt the wavelength of 510 nm as the determination of Fe(VI) solution [14]. Molar absorption coefficient of Fe(VI) solution is 1150 M^−1^·cm^−1^ at 510 nm wavelength [15]. In addition, due to the Fe(VI) easily decomposed to Fe(OH)_3_, interfering with the detection results, samples were measured after centrifugation at 6000 r·min^−1^ for 10 min, and the supernatant was taken for spectrophotometry.

#### 2.3.2. Detection of Three Kinds of Sulfa Antibiotics by HPLC

High performance liquid chromatography (HPLC) was adopted on three kinds of sulfa antibiotics for quantitative analysis. The concentrations of sulfadiazine, sulfamerazine, and sulfamethoxazole were measured by Waters e2695-2489 HPLC (UV detector) and Waters Symmetry C18 column to column (250 mm × 4.6 mm). Acetonitrile and mass fraction of 0.1% formic acid solution, the volume ratio of 40 : 60, were adopted for mobile phase, with flow rate 0.8 mL·min^−1^, column temperature 35°C, and detection wavelength 270 nm. Under these conditions, sulfadiazine, sulfamerazine, and sulfamethoxazole peaked well and the peak time was 4.081 min, 4.429 min, and 6.015 min, respectively.

#### 2.3.3. Detection of Three Kinds of Sulfa Antibiotics and Their Degradation Product by LC-MS-MS

HPLC-MS (Waters e2695 Separation Module Thermo Finnigan TSQ Quantum) was adopted for qualitative analysis of sulfadiazine, sulfamerazine, sulfamethoxazole, and their products; HPLC analysis conditions were as follows: chromatographic column was C18 column (Thermo Basic C18, 150 mm × 2.1 mm), mobile phase was acetonitrile, and the mass fraction was 0.1% formic acid solution, using gradient elution mode. Detection time lasted for 30 min, mobile phase flow rate was 300 *μ*L·min^−1^, the column temperature was 35°C, and the injection volume was 10 *μ*L.

MS conditions were as follows: full scan mode was employed for analysis of sulfadiazine, sulfamerazine, sulfamethoxazole, and their products; MS spectrometry ionization source was heating type electrospray ionization source (H-ESI), with specific parameters shown in Table 2.

These three sulfa antibiotics could be separated and peaked well in this full scale mode; the peak time of sulfadiazine, sulfamerazine, and sulfamethoxazole was 3.05 min, 4.87 min, and 13.13 min, respectively.

## 3. Results and Analysis

### 3.1. Fe(VI)-US Synergy

**Figure 2:**
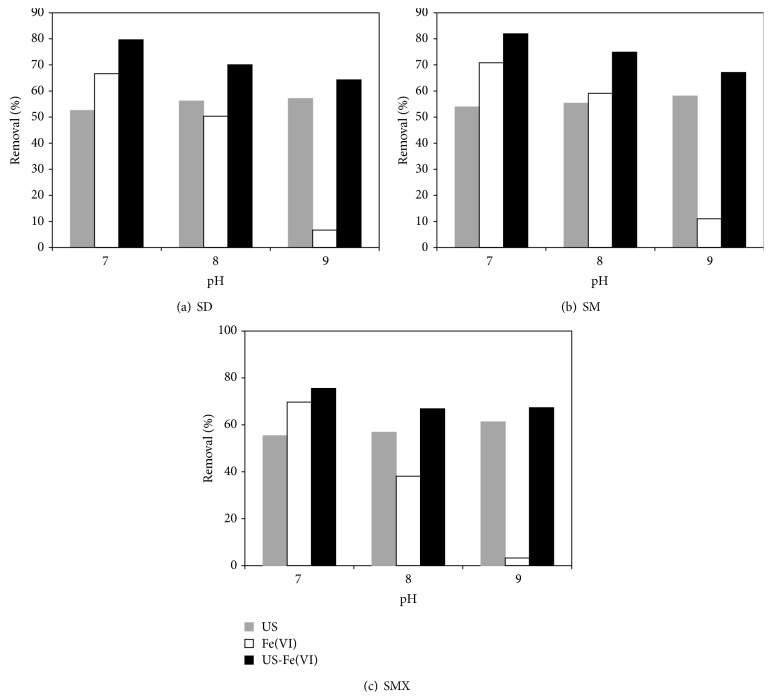
The removal effect of sulfa antibiotics oxidized by Fe(VI)-US: (a) sulfadiazine; (b) sulfamerazine; (c) sulfamethoxazole.

The initial concentrations of sulfadiazine, sulfamerazine, and sulfamethoxazole were all 0.02 mmol·L^−1^, the ultrasonic frequency was 800 kHz, the output electric power was 100 W, reaction pH was controlled ranging from 7 to 9, and 0.05 mmol·L^−1^ of potassium ferrate was added to the reaction solution, studying the removal effect of sulfa antibiotics by Fe(VI)-US.  Figure 2 presented the removal effect of sulfa antibiotics by ultrasound (US), potassium ferrate (Fe(VI)), and potassium ferrate-ultrasound (Fe(VI)-US), respectively, at different pH values at the reaction time of 10 min; results showed that, compared with the degradation by US or Fe(VI) alone, Fe(VI)-US had a significant role in promoting degradation of sulfa antibiotics. It was demonstrated that ultrasonic irradiation had a good effect for degradation of oxidation of sulfadiazine, sulfamerazine, and sulfamethoxazole, and the common oxidation of ultrasonic irradiation and potassium ferrate greatly increased the removal rate of sulfa antibiotics, which might be one of the reasons why Fe(VI)-US technology had synergistic effect.

#### 3.1.1. Promotion Effect of Fe^3+^

In the process of oxidizing sulfa antibiotics by potassium ferrate, Fe(VI) turned to Fe(III) finally; the other reason why Fe(VI)-US had synergy might be that the existence of Fe(III) promoted the degradation of sulfa antibiotics. Fe^3+^ was added into reaction solution, studying the effect of Fe^3+^ on degradation of sulfa antibiotics by ultrasonic irradiation, as shown in Table 3. According to the experimental data, Fe^3+^ had a promoted effect on degradation of sulfa antibiotics by ultrasonic irradiation at the pH of 7–9. The reaction rate increased continuously and the removal effect was obvious with Fe^3+^ dosage increasing from 0.00 mmol·L^−1^ to 0.10 mmol·L^−1^. However, this promotion decreased slightly when Fe^3+^ dosage reached up to 0.20 mmol·L^−1^.

There might be two reasons for Fe^3+^ promoting on degradation of sulfa antibiotics by ultrasonic irradiation. First, the addition of Fe^3+^ increases the ionic strength. Studies [16] have shown that, with the increase of ionic strength, more and more water molecules tend to be combined with anion and cation forming hydration film, making water molecules dissolving organic matters reduced, which results in decrease of the solubility of organic matters in water, more advantageous to head toward the cavitation bubble of gas-liquid interface migration in ultrasonic field. Cavitation bubble of gas-liquid interface is the active center of ultrasonic chemical reaction, with the presence of high concentrations of hydroxyl radicals and supercritical water layer in this region [17]; thus, it is more conducive for reaction. However, with the increase of ionic strength in water, the saturated vapor pressure of water decreases, while the surface tension increases, causing the sound pressure for cavitation increasing; the number of cavitation bubbles formed within a unit time decreases, cavitation weakens, and the removal effect lowers.

Second, the water molecules under ultrasonic cavitation will enter cavitation bubble then to form ·OH and ·H via internal thermal cracking. A lot of ·OH quickly recombine and generate H_2_O_2_ at a relatively low temperature of cavitation bubbles of gas-liquid interface; H_2_O_2_ eventually spreads into the main body solution and performs class Fenton reaction with Fe^3+^ ((3) and (4)) to generate more ·OH, thus promoting the removal of sulfa antibiotics as follows:

(3) $$\begin{array}{c} \text{F}{\text{e}}^{3+}+{\text{H}}_{2}{\text{O}}_{2}⟶\text{F}{\text{e}}^{2+}+\text{H}{\text{O}}_{2}\cdot +{\text{H}}^{+} \end{array}$$

(4) $$\begin{array}{c} \text{H}{\text{O}}_{2}\cdot +{\text{H}}_{2}{\text{O}}_{2}⟶{\text{O}}_{2}+{\text{H}}_{2}\text{O}+\cdot \text{OH} \end{array}$$

### 3.2. Effects of Degradation of Sulfa Antibiotics by Fe(VI)-US

#### 3.2.1. Effect of pH

**Figure 3:**
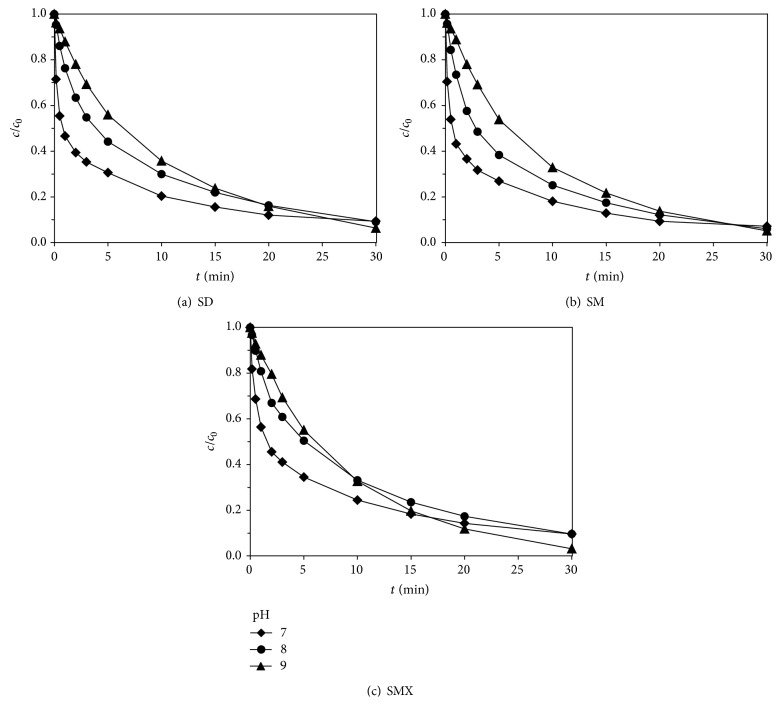
Effect of pH values on degradation of sulfa antibiotics by Fe(VI)-US: (a) sulfadiazine; (b) sulfamerazine; (c) sulfamethoxazole.

The pH of reaction solution was adjusted by buffer solution; pH value was ranging from 7 to 9. The initial concentrations of sulfadiazine, sulfamerazine, and sulfamethoxazole were all 0.02 mmol·L^−1^, the ultrasonic frequency was 800 kHz, the output electric power was 100 W, reaction pH was controlled at 7–9, and 0.05 mmol·L^−1^ of potassium ferrate was added into the reaction solution, studying the effect of pH on degradation of sulfa antibiotics by Fe(VI)-US.  Figure 3 showed the effect of different pH values on degradation of sulfadiazine, sulfamerazine, and sulfamethoxazole by Fe(VI)-US.

As shown in Figure 3, the reaction rate declined with the increasing pH value. The degradation rate of these three sulfa antibiotics was the fastest at pH 7, and the removal rate reached a maximum at the first 10 min. As reaction extended, when the reaction time was 30 min, the removal rate of all these three sulfa antibiotics reached maximum at pH 9. According to the study of sulfa antibiotics oxidized by potassium ferrate, oxidation rate was fast, and reaction was basically completed in the first 2 min when the pH was in neutral or acidic conditions. Due to the oxidation of potassium ferrate dominated at the early stage of Fe(VI)-US, the effect of pH on degradation of sulfa antibiotics by Fe(VI)-US was consistent with that of potassium ferrate. The oxidation of ultrasonic irradiation took leading role at the late stage; thus, the effect of pH was consistent with degradation of sulfa antibiotics by ultrasonic irradiation.

#### 3.2.2. Effect of Potassium Ferrate Dosages

**Figure 4:**
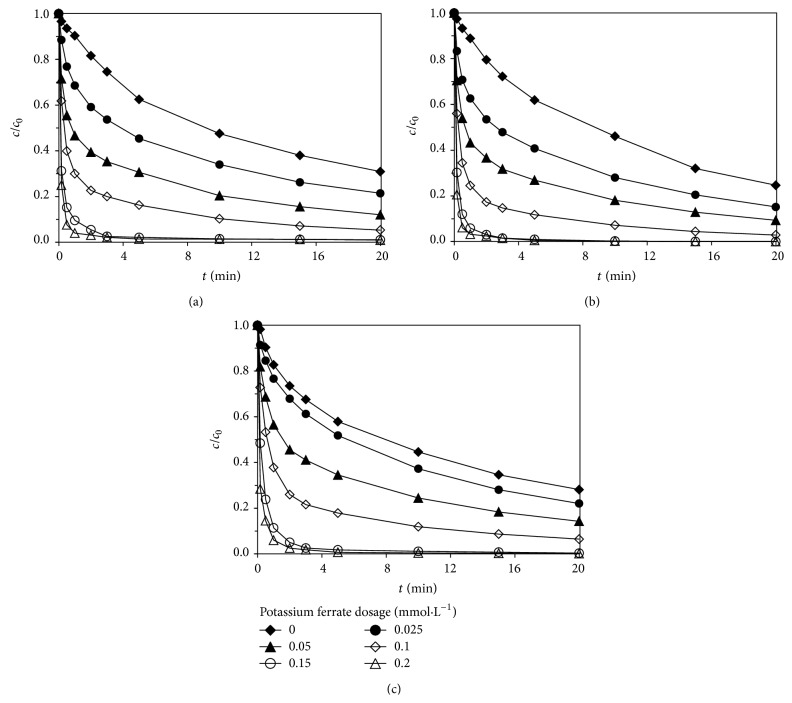
Effect of potassium ferrate dosages on degradation of sulfa antibiotics by Fe(VI)-US: (a) sulfadiazine; (b) sulfamerazine; (c) sulfamethoxazole.

Different concentrations of potassium ferrate solution was added into the reaction solution, studying the effect of potassium ferrate dosages on degradation of sulfa antibiotics by Fe(VI)-US; the experimental results are shown in Figure 4.

The potassium ferrate dosages ranged from 0.00 mmol·L^−1^ to 0.20 mmol·L^−1^, the initial concentrations of sulfadiazine, sulfamerazine, and sulfamethoxazole were all 0.02 mmol·L^−1^, the ultrasonic frequency was 800 kHz, the output electric power was 100 W, and reaction pH was controlled at about 7. Figure 4 showed that potassium ferrate dosages had a significant effect on degradation of sulfa antibiotics by Fe(VI)-US; with the increase of potassium ferrate dosages, the degradation rate of sulfa antibiotics by Fe(VI)-US increased. When potassium ferrate dosage was 0.20 mmol·L^−1^, all these three sulfa antibiotics were removed quickly, with the degradation rate reaching up to more than 98% at reaction time of 5 min. The increase of potassium ferrate dosages enhanced the leading role of oxidation of potassium ferrate in Fe(VI)-US; thus the effect of potassium ferrate dosages on degradation of sulfa antibiotics was consistent with that of oxidized by potassium ferrate.

#### 3.2.3. Effect of Ultrasonic Frequencies

**Figure 5:**
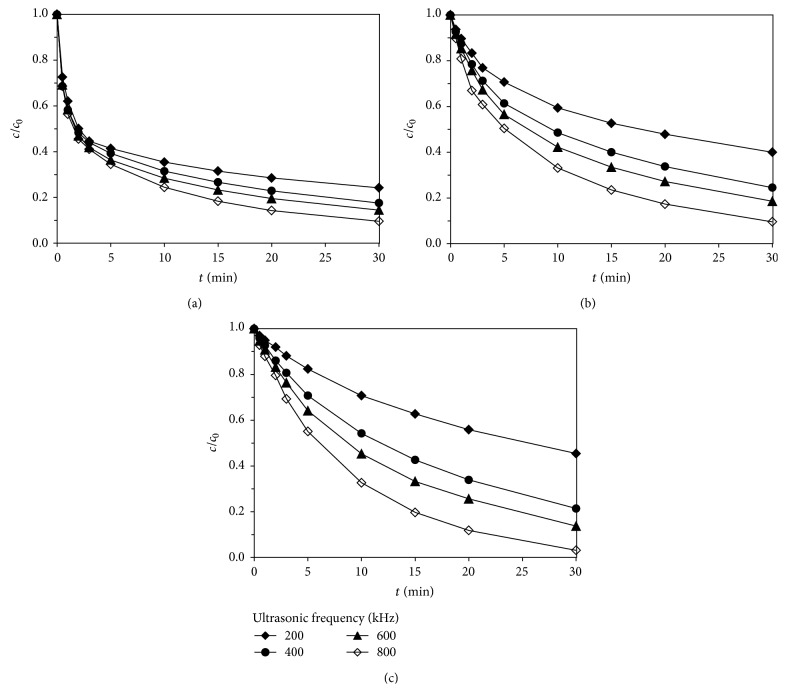
Effect of ultrasonic frequencies on degradation of sulfamethoxazole by Fe(VI)-US: (a) pH = 7; (b) pH = 8; (c) pH = 9.

Ultrasonic frequency in experiments was 200, 400, 600, and 800 kHz, respectively, the initial concentration of sulfamethoxazole was 0.02 mmol·L^−1^, and the output electric power was 100 W, studying the effect of ultrasonic frequency on degradation of sulfa antibiotics by Fe(VI)-US.  Figure 5 showed the effect of ultrasonic frequency on degradation of sulfa antibiotics by Fe(VI)-US in different pH conditions.

In the study of degradation of sulfa antibiotics by ultrasonic irradiation, the removal rate of sulfa antibiotics increased with the increase of ultrasonic frequency. The increase of ultrasonic frequency increased the number of hydroxyl radicals by ultrasonic cavitation and, in return, strengthens the Fe(VI)-US synergy, promoting the reaction. In addition, the oxidation rate of potassium ferrate decreased as the pH value rose. The higher the pH was, the stronger Fe(VI)-US the synergy was, and the amplitude of degradation of sulfa antibiotics increased with the increase of ultrasonic frequency.

#### 3.2.4. Effect of Ultrasonic Powers

**Figure 6:**
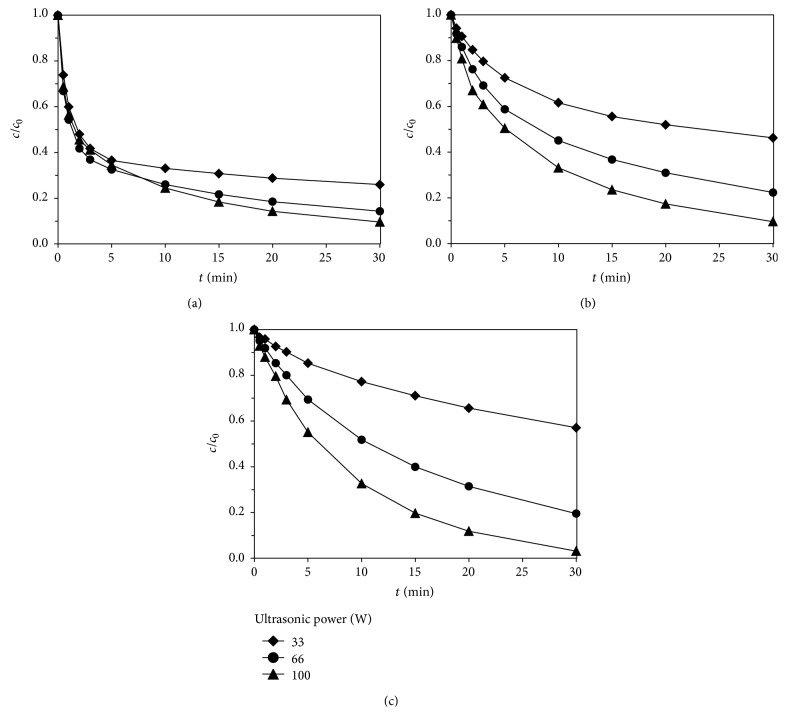
Effect of ultrasonic powers on degradation of sulfamethoxazole by Fe(VI)-US: (a) pH = 7; (b) pH = 8; (c) pH = 9.

Ultrasonic power was regulated, respectively, to 33, 66, and 100 W, studying the effect of ultrasonic powers on degradation of sulfa antibiotics by Fe(VI)-US. The initial concentration of sulfamethoxazole was 0.02 mmol·L^−1^, the ultrasonic frequency was 800 kHz, the effect of ultrasonic powers on degradation of sulfa antibiotics under different pH conditions was shown in Figure 6. According to the experimental data, the effect of ultrasonic powers on degradation of sulfa antibiotics by Fe(VI)-US was consistent with that of ultrasonic frequency, and the degradation rate of sulfamethoxazole increased with the increase of ultrasonic powers. Moreover, the degradation rate increased more obviously at higher pH values.

The number of cavitation bubbles produced increased per unit with the increase of ultrasonic powers, so did the cavitation effect, and the Fe(VI)-US synergy was enhanced at the same time. Oxidation rate of potassium ferrate declined with the increase of pH, and the synergy of Fe(VI)-US was more obvious with higher pH values. The larger the ultrasonic power was, the more significant the amplitude of degradation effect was.

### 3.3. Mechanism of Degradation of Sulfa Antibiotics by Fe(VI)-US

**Figure 7:**
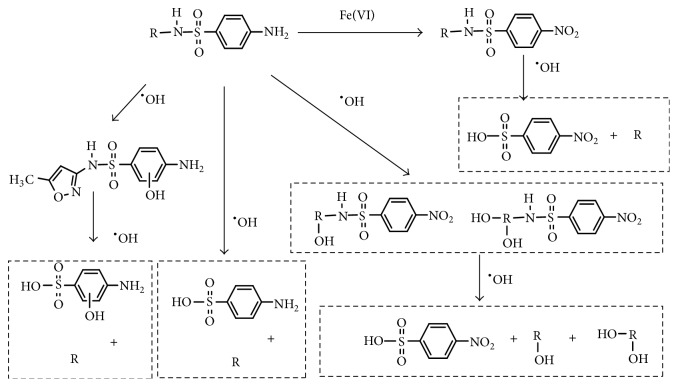
Proposed degradation pathway of sulfa antibiotics oxidized by potassium ferrate combined with ultrasound.

The main identified intermediates of sulfa antibiotics oxidized by Fe(VI)-US were analyzed by LC-HESI-MS-MS, main products of sulfadiazine, sulfamerazine, and sulfamethoxazole were listed in Table 4, and the results showing that degradation of these three sulfa antibiotics by Fe(VI)-US were all removed by hydroxyl radicals with oxidation. According to the analysis of products, the degradation pathway diagram of sulfa antibiotics oxidized by Fe(VI)-US was made, and the specific degradation process was shown in Figure 7.

## 4. Conclusions

All of sulfadiazine, sulfamerazine, and sulfamethoxazole could be degraded by ultrasound well, and the reaction process was in accordance with pseudo-second order reaction kinetics. As ultrasonic irradiation time extended, the degradation rate of sulfa antibiotics increased continuously, and the removal rate of sulfadiazine, sulfamerazine, and sulfamethoxazole reached 77.46%, 82.46%, and 82.46% after reaction time 30 min, respectively.

Compared with the degradation of sulfa antibiotics oxidized by potassium ferrate or ultrasonic irradiation, Fe(VI)-US technology has a significant role in promoting the degradation of sulfa antibiotics. The synergy of Fe(VI)-US mainly includes two aspects: one is the common oxidation of potassium ferrate and ultrasound, and the other is that Fe(VI) finally turns into Fe(III) in the process of degradation of sulfa antibiotics oxidized by potassium ferrate, and the existence of Fe(III) promotes the removal effect of sulfa antibiotics by ultrasound.

Degradation of sulfa antibiotics by Fe(VI)-US are influenced by different sulfa antibiotics, pH values, potassium ferrate dosages, ultrasonic frequencies, and ultrasonic powers, and orthogonal experiments are performed to study the above five factors on degradation of sulfa antibiotics and the influence of primary and secondary order of reaction rate. The effects of pH values, potassium ferrate dosages, and sulfa antibiotics type on the reaction rate are the most significant, with the pH values being the maximum. Thus, pH values and sulfa antibiotics dosages should be controlled reasonably in experiments in order to ensure economy while achieving optimal removal effect.

## Figures and Tables

**Table 1:** The physical and chemical properties of standards.

Substance name	Formula	Structure	Molecular weight/Da.	pK_*a*_	
				pK_*a*1_	pK_*a*2_
Sulfadiazine	C_10_H_10_N_4_O_2_S	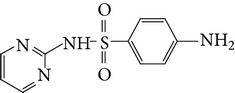	250.27	2.49	6.50

Sulfamerazine	C_11_H_12_N_4_O_2_S	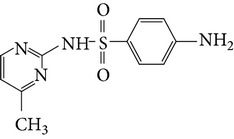	264.30	—	7.00

Sulfamethoxazole	C_10_H_11_N_3_O_3_S	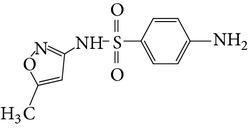	253.28	1.74	5.70

**Table 2:** Mass spectrometric parameters in full scan mode.

Parameter name		Parameter values
Electrospray voltage		3500 V
Sheath gas	N_2_	0.28 MPa (40 psi)
Auxiliary gas	N_2_	0.07 MPa (10 psi)
Ion transport capillary temperature		270°C
Scanning range		50~500 *m/z *

**Table 3:** Effect of Fe^3+^ on ultrasonic degradation of sulfa antibiotics.

Substance name	pH	Ultrasound		Fe^3+^-Ultrasound		
		*k*/L·mmol^−1^·min^−1^	*R* ^2^	Fe^3+^ dosage/mmol·L^−1^	*k*/L·mmol^−1^·min^−1^	*R* ^2^
Sulfadiazine	7	5.6666	0.9992	0.05	6.2572	0.9954
	8	6.9630	0.9922	0.05	7.6543	0.9882
	9	8.2422	0.9906	0.05	9.7026	0.9623

Sulfamerazine	7	7.8615	0.9961	0.05	9.3386	0.9810
	8	8.2518	0.9913	0.05	10.371	0.9744
	9	9.9267	0.9874	0.05	11.886	0.9628

Sulfamethoxazole	7	6.7640	0.9930	0.05	7.1840	0.9899
	8	7.8218	0.9901	0.05	8.4047	0.9801
	9	9.8719	0.9910	0.05	12.358	0.9817
	7	6.7640	0.9930	0.10	7.3010	0.9819
	8	7.8218	0.9901	0.10	9.0281	0.9814
	9	9.8719	0.9910	0.10	14.884	0.9647
	7	6.7640	0.9930	0.20	7.2641	0.9922
	8	7.8218	0.9901	0.20	8.4559	0.9781
	9	9.8719	0.9910	0.20	13.590	0.9822

**Table 4:** Main identified intermediates of sulfa antibiotics.

Substance name	*m*/*z*	Structure	*m*/*z*	Structure
Sulfadiazine	267	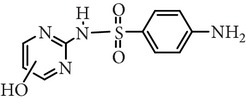	267	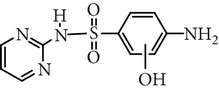
	173	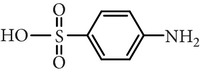	96	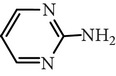
	281	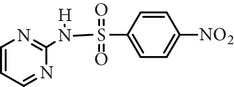		

Sulfamerazine	281	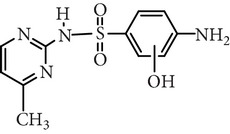	281	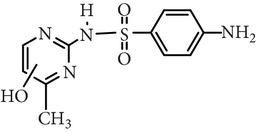
	173	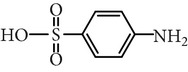	110	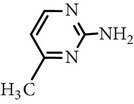
	295	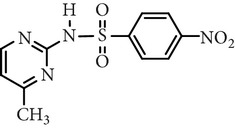		

Sulfamethoxazole	270	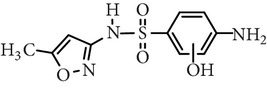	270	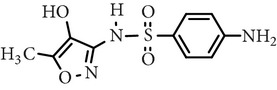
	288	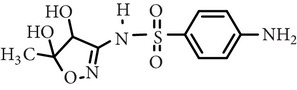		
	173	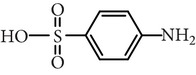	99	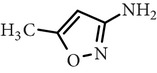
	284	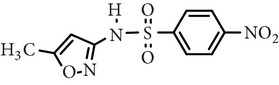	270